# An antioxidant therapy elicits distinct transcriptome responses in 22q11-deleted upper layer cortical projection neurons

**DOI:** 10.1242/dmm.052786

**Published:** 2026-09-09

**Authors:** Shah Rukh, Daniel W. Meechan, Abra Roberts, Connor Siggins, Zachary D. Erwin, Thomas M. Maynard, Anthony-S. LaMantia

**Affiliations:** ^1^ Center for Neurobiology Research, The Fralin Biomedical Research Institute at Virginia Tech-Carilion School of Medicine, Roanoke, VA 24016, USA; ^2^ Translational Biology, Medicine and Health Program, Virginia Tech-Carilion School of Medicine, Roanoke, VA 24016, USA; ^3^ Virginia Tech-Carilion School of Medicine, Roanoke, VA 24016, USA; ^4^ Department of Biological Sciences, Virginia Tech, Blacksburg, VA 24016, USA

**Keywords:** Development, Antioxidant therapy, Copy number variants, Mitochondrial function, 22q11 deletion, Cortical development

## Abstract

We characterized *in vitro* and *in vivo* responses to the antioxidant N-acetyl cysteine (NAC) in mice that model the heterozygous deletion of genes at human chromosome 22q11.2 associated with the 22q11.2 deletion syndrome (22q11DS). In this ‘large deletion’ (*LgDel*) mouse model, NAC restores the growth and connectivity of developing upper layer cortical projection neurons (L 2/3 PNs) and improves their cognitive performance. It ameliorates L 2/3 PN developmental pathology without restoring wild-type (WT) growth patterns or expression levels of downstream targets of 22q11-deleted genes. Instead, neuronal growth and antioxidant defense genes are differentially expressed when comparing *LgDel* and WT PNs, i.e. some are generally regulated by NAC, while others are responsive only in the context of 22q11 deletion. NAC also elicits the expression of genes involved in growth and antioxidant defense in differentiating 22q11-deleted L 2/3 PNs in postnatal *LgDel* mouse cortex rather than restores 22q11 downstream targets to WT levels; however, these L 2/3 PN-selective *in vivo* changes differ substantially from those in primary culture. Thus, the therapeutic response to NAC that diminishes oxidative stress-related L 2/3 PN developmental circuit and behavioral pathology due to 22q11 deletion has a distinct *in vivo* signature.

## Teaser

**Summary:** Antioxidant therapy circumvents 22q11 deletion-dependent gene expression changes in cortical neurons by establishing divergent *in vitro* vs *in vivo* growth and transcriptional states that do not resemble wild-type states *in vitro* or *in vivo*.

## INTRODUCTION

Therapies for neurodevelopmental disorders (NDDs) with known genetic causes have focused on restoring single-mutant gene function in monogenic disorders or one or more mutant genes that contribute to polygenic NDD risk (Berry-Kravis et al., 2018; Chen and Geschwind, 2022; Devinsky et al., 2025; Lauffer et al., 2024; Sandweiss et al., 2020; Zylka, 2020). Nevertheless, substantial challenges in targeting, timing and regulation of functional copies of mutant genes or RNA surrogates remain (Bajikar et al., 2024). Alternatively, downstream genes or networks disrupted by NDD-associated mutations can be potential therapeutic targets, but aligning these targets with effective therapeutic agents remains difficult (Copping et al., 2021; Diaz-Caneja et al., 2021; Paul and Potter, 2024; Pedini et al., 2023). To determine whether restoring gene expression altered by a causal NDD mutation is necessary for therapeutic efficacy, we asked whether N-acetyl cysteine (NAC), an antioxidant that reverses developmental pathology of upper layer (layers 2/3) cortical projection neurons (L 2/3 PNs) – a key feature of multiple NDDs (Batiuk et al., 2022; Lewis and Sweet, 2009; Parikshak et al., 2013; Sacai et al., 2020) – and related behavioral deficits in the 22q11.2 deletion syndrome *LgDel* mouse model (22q11; Meechan et al., 2015a) also restores L 2/3 PN cellular and transcriptional states altered by 22q11 deletion or whether it elicits a divergent therapeutic response, independent of 22q11 gene dosage-mediated changes in L 2/3 PNs *in vitro* as well as *in vivo*.

22q11.2 deletion syndrome (22q11DS; also known as DiGeorge syndrome) is caused by a microdeletion on the long arm of chromosome 22 and is one of the most common polygenic NDD syndromes. It also carries one of the most robust genetic risks for schizophrenia, autistic spectrum disorder and other behavioral pathologies. Thus, therapies that target specific neurons and circuits compromised in 22q11DS may provide a template for interventions in a broader range of NDDs. It is unclear, however, how to integrate mechanisms inferred from *in vitro* assays with *in vivo* actions of potential therapeutic agents in 22q11DS or other NDDs. Indeed, discrete therapeutic targets have yet to be identified, even in induced pluripotent stem cells (iPSCs) from 22q11DS patients, due to challenges generating neurons or glia for which changes resemble established 22q11DS cellular pathology (Fiksinski et al., 2023; LaMarca et al., 2018; Lin et al., 2016; Rao et al., 2025). We have shown previously in the *LgDel* mouse that NAC selectively reverses oxidative stress in L 2/3 PNs *in vitro* and *in vivo*, by diminishing elevated reactive oxygen species (ROS) and reversing their impact on L2/3 PN growth as well as cytological and synaptic integrity (Fernandez et al., 2019). It is unclear, however, whether NAC returns 22q11-deleted L 2/3 PNs to typical growth or transcriptional states. Indeed, NAC may establish a divergent ‘therapeutic’ state through cellular, metabolic or transcriptional responses that circumvent changes due to decreased expression of heterozygously deleted 22q11.2 genes – hereafter referred to as ‘diminished 22q11 gene dosage’.

We assessed growth, metabolic and transcriptional states of developing wild-type (WT) and *LgDel* L 2/3 PNs with and without NAC treatment *in vitro* and *in vivo*, as well as their states that reflect a related heterozygous mutation of *Txnrd2*, a key 22q11-deleted mitochondrial gene associated with ROS-mediated NAC-sensitive L 2/3 PN pathology. NAC ameliorates but does not directly reverse growth, metabolic and transcriptional disruption due to diminished 22q11 gene dosage in differentiating L 2/3 PNs. Our results indicate that circumventing changes due to aberrant cellular states, such as oxidative stress due to elevated ROS, rather than directly restoring mutant/downstream gene function can provide significant – and perhaps more readily achievable – therapeutic benefits in complex NDDs like 22q11DS.

## RESULTS

### A distinct transcriptional state defines 22q11-deleted L 2/3 PNs *in vitro*

**Fig. 1.**
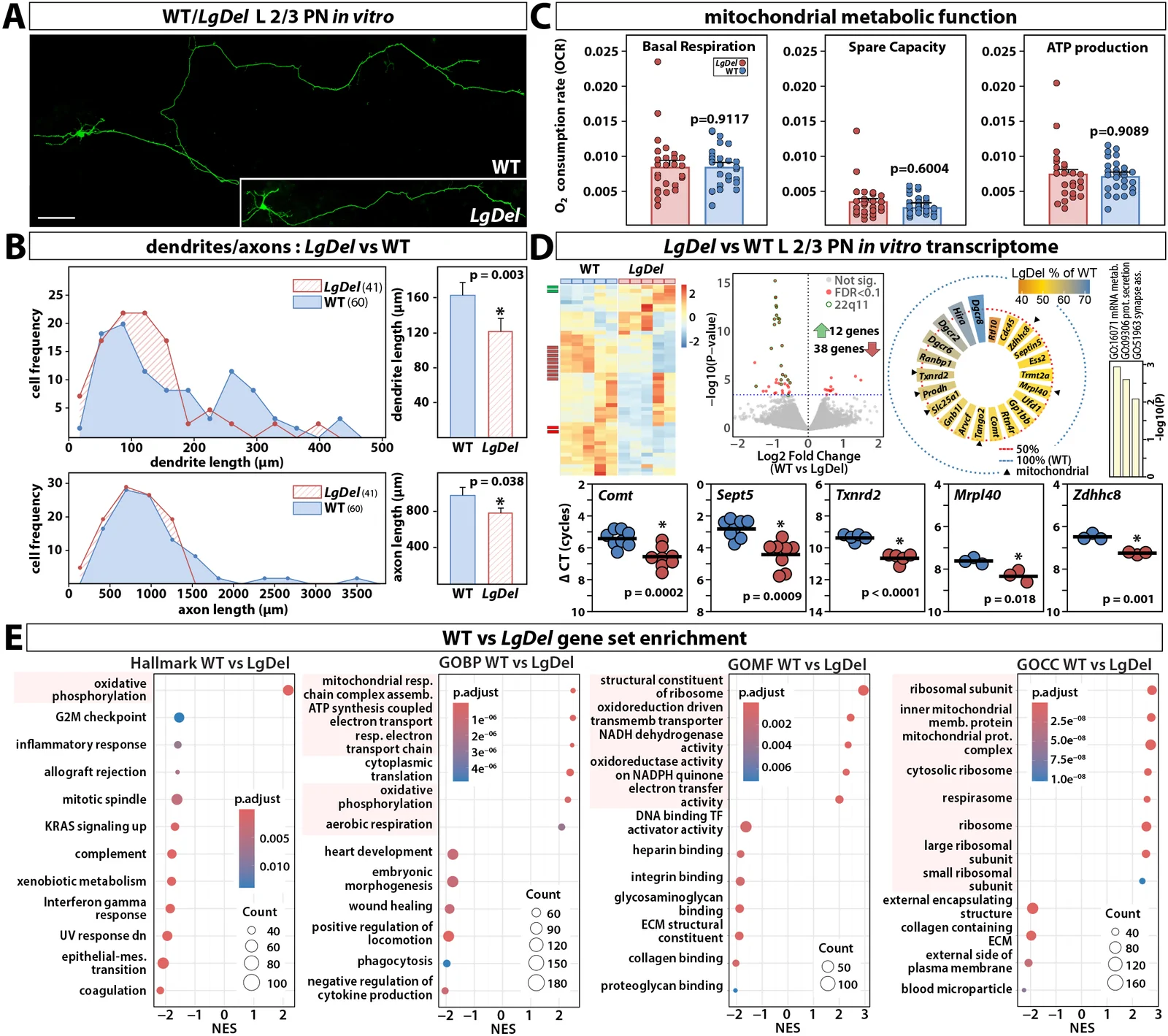
**Distinct metabolic and transcriptional states accompany selective dendrite and axon growth changes due to 22q11 gene deletion in mouse primary L 2/3 PNs *in vitro*.** (A) Microscopy image showing WT (main image) and *LgDel* (inset) L 2/3 PNs *in vitro* with larger vs smaller dendrite and axon arbors. The neuron and its processes are labeled via expression of eGFP reporter plasmid (green; scale bar: 50 µm). (B) Frequency distribution of WT and *LgDel* L 2/3 PNs *in vitro* based upon total dendrite (top, left) or axon (bottom, left) length (*n*=F55 WT, 36 *LgDel* L 2/3 PNs; one-way ANOVA, Holm−Šídák) and mean differences in total dendrite (top, right) and axon lengths (bottom, right). (C) Basal respiration, spare capacity and ATP production in *LgDel* vs WT littermate L 2/3 PNs *in vitro* based upon O_2_ consumption (Seahorse; *n*=19 *LgDel*, 16 WT, multi-well cultures/four litters; one-way ANOVA, Holm−Šídák). (D) WT vs LgDel L 2/3 PNs *in vitro* transcriptome comparison (five biological replicates of pooled L 2/3 PN samples; *n*=15 fetuses/six litters WT; 15 fetuses/seven litters *LgDel*) identifies 50 differentially expressed genes (DEGs) in LgDel L 2/3 PNs (FDR<0.1), including 21 22q11-deleted orthologues (top, right). Gene ontology (GO) analysis of 19 non-22q11-deleted genes (top, far right) indicates DEGs enriched for metabolic and neuron-specific pathways. qPCR of WT and *LgDel* RNA L 2/3 PNs *in vitro* samples confirms 50% diminished expression of 5 22q11-deleted genes (bottom). (E) Hallmark gene set enrichment analysis (GSEA) and GOs for biological processes (GOBP), molecular functions (GOMF) and cellular compartments (GOCC) identify oxidative phosphorylation and mitochondrial function gene sets in WT vs *LgDel* L 2/3 PNs.

L 2/3 PNs *in vitro* obtained from the cortex of *LgDel* mice at embryonic day (E)16.5 provide a uniform population with pathological changes similar to those in *LgDel* L 2/3 PNs *in vivo* (Fernandez et al., 2019) for cellular and transcriptome comparison. We confirmed limited *LgDel* L 2/3 PNs *in vitro* growth (Fig. 1) and assessed modes of growth based upon total dendrite or axon length per cell. WT L 2/3 PN *in vitro* dendritic arbors were bimodally distributed (Fig. 1A,B, top; see also Fig. 3). In contrast, *LgDel* L 2/3 PNs *in vitro* dendritic arbors were distributed around a single mean that overlaps with the smaller WT population. Similarly, the *LgDel* L 2/3 PN *in vitro* axon distribution (Fig. 1B, bottom) lacked the WT ‘tail’ of larger axons. Finally, a modest but significant correlation between dendrite and axon length for individual WT L 2/3 PNs *in vitro* [coefficient of determination (r^2^)=0.1345, *P*=0.004] (Fig. S1) is lost in *LgDel* (r^2^=0.0065, *P*=0.616). Thus, 22q11 gene deletion either eliminates a L 2/3 PN *in vitro* neuroblast population with maximal dendrite and axon growth capacity or limits growth of a subset of L 2/3 PNs *in vitro*.

We next asked whether growth-restricted *LgDel* vs WT L 2/3 PNs *in vitro* have altered mitochondrial function based upon O_2_ consumption. We found no significant differences in three mitochondrial metrics: basal respiration, spare capacity and ATP production (Fig. 1C). *LgDel* L 2/3 PNs *in vitro* retain some typical metabolic functions, despite limited dendrite and axon growth and elevated ROS (Fernandez et al., 2019). Typical O_2_ consumption in 22q11-deleted L 2/3 PNs *in vitro* may reflect compensatory mechanisms that stabilize energy production despite increased oxidative stress via changes in cellular redox states that facilitate mitochondrial function and improve ROS handling similar to those reported previously for other cell types (Panieri and Santoro, 2016; Shadel and Horvath, 2015; Zhang et al., 2022).

A distinct transcriptional state accompanies selectively restricted growth and apparent mitochondrial homeostasis in *LgDel* L 2/3 PNs *in vitro* (Fig. 1D, top row, left). Identified were 50 differentially expressed genes (DEGs), i.e. genes showing a statistically significant difference in expression levels [false discovery rate (FDR)<0.1], with expression of 38 genes reduced and of 12 genes enhanced (Fig. 1D, top row, center). As expected, 21 genes that are deleted within 22q11, including six transcripts that encode proteins localized to mitochondria (Campbell et al., 2023; Maynard et al., 2008; Motahari et al., 2019), are among the 38 DEGs with reduced expression levels. Quantitative PCR (qPCR) of a 22q11 gene subset confirms the 50% decrease in L 2/3 PNs *in vitro* (Fig. 1D, bottom row). The 17 remaining diminished DEGs include the following: *Nyap2* (Yokoyama et al., 2011) and *Gucy1a2* (Haase et al., 2010) associated with neuron growth; *Pclo* and *Thbs2* (Mazur et al., 2021; Waites et al., 2013), implicated in synapse formation and maintenance; *Proser1* (Fleming et al., 2024), a chromatin regulator; *Lnpep* (Zhang et al., 2017), a contributor to synaptic plasticity; and *Vps13c* (Monfrini et al., 2022; Schröder et al., 2024), implicated in mitochondrial quality control and lipid transfer between endoplasmic reticulum and mitochondria. Significantly enhanced transcripts include *Emc10*, a regulator of neural development (Shao et al., 2021) that has previously been associated with 22q11 deletion (Thakur et al., 2025); *Aopep*, which modulates synaptogenesis (Garavaglia et al., 2022); and *B9d2*, which is associated with signal transduction by cilia (Caenen-Braz et al., 2024). Gene ontology (GO) analysis indicated that DEGs engage mRNA metabolism, protein secretion and synapse assembly gene sets (Fig. 1D, top, far right). qPCR confirmed differential expression of *Nyap2* (see Fig. 3); however, two other DEGs, *Lnpep* and *Gucy1a2* did not differ significantly. Thus, DEGs that modulate bioenergetic homeostasis, neuron or synaptic differentiation change in register with altered growth and differentiation in 22q11-deleted L 2/3 PNs *in vitro*.

To further analyze divergence between WT (20,650 transcripts) and *LgDel* (25,200 transcripts) transcriptomes L 2/3 PNs *in vitro*, we used gene set enrichment analysis (GSEA) (Fig. 1E, top left) (Subramanian et al., 2005). The most significantly positive enriched hallmark gene set in *LgDel* L 2/3 PNs *in vitro* is that of oxidative phosphorylation, indicating altered expression of mitochondrial energy metabolism genes in these neurons, which we have previously shown to have increased levels of mitochondrial and cytoplasmic ROS (Fernandez et al., 2019). Additional GSEA identified significant positive enrichment in metabolism-related gene sets (Fig. 1E), matched by depleted gene sets for cytoskeletal dynamism, cell adhesion and motility. Apparently, 22q11-deleted L 2/3 PNs *in vitro* acquire transcriptional states that support metabolic and mitochondrial homeostasis while limiting neuronal growth and differentiation.

### Constitutive heterozygous *Txnrd2* deletion and L 2/3 PN *in vitro* growth regulation

**Fig. 2.**
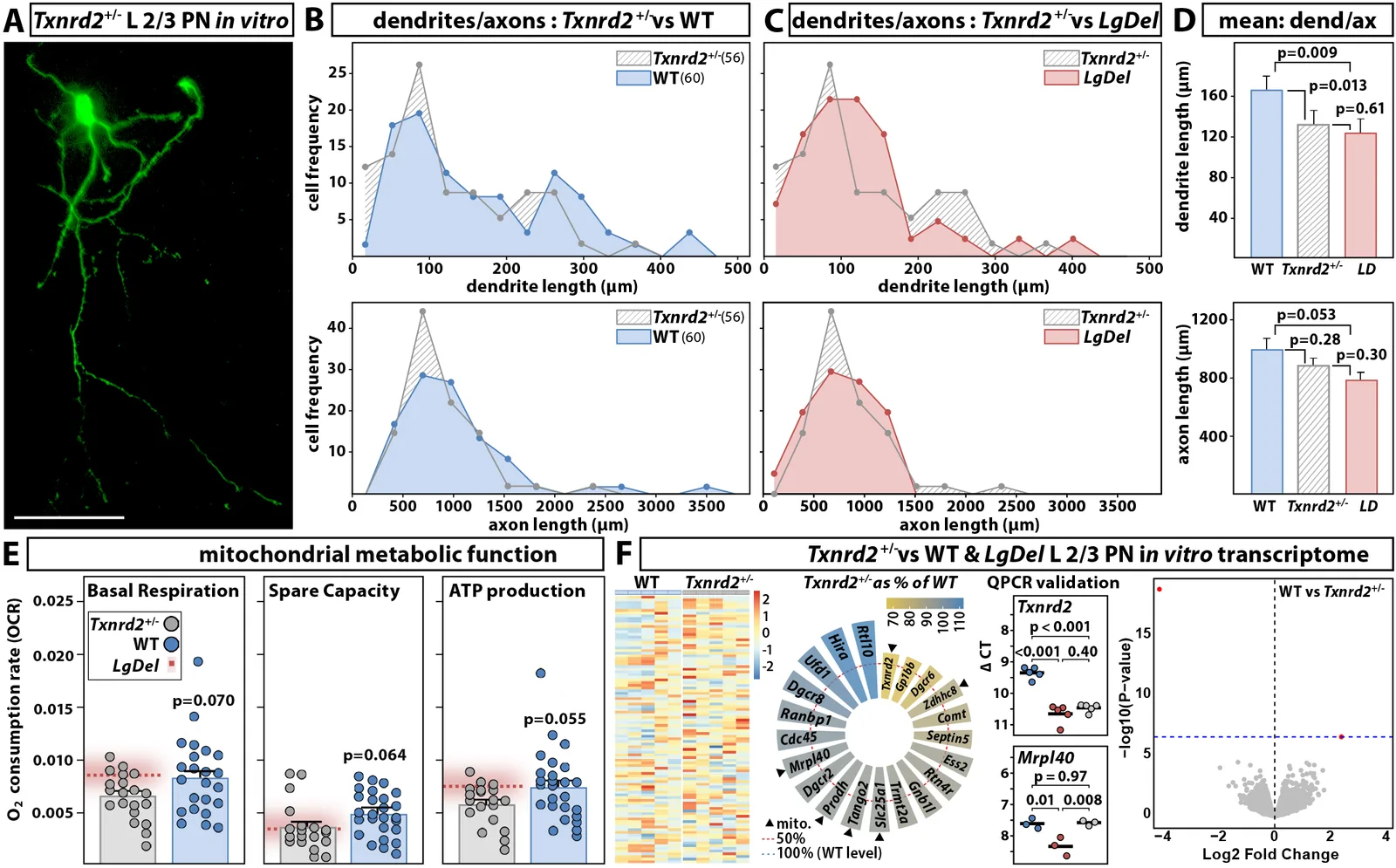
**Distinct growth, metabolic and transcriptional responses of *Txnrd2^+/−^* L 2/3 PNs *in vitro*.** (A) Microscopy image showing an L 2/3 PN *in vitro* from a cortical neuroblast harvested from an E16.5 *Txnrd2^+/−^* fetus. The neuron and its processes are labeled via expression of eGFP reporter plasmid (green; scale bar: 50 µm). (B) Bimodal frequency distributions of smaller vs larger dendrite arbors in WT and *Txnrd2*^+/−^ L 2/3 PNs *in vitro*. The *Txnrd2*^+/−^ distribution is shifted slightly leftward. (C) Similar distributions of *Txnrd2*^+/−^ and WT axon lengths accompany increased smaller axon and diminished larger axon frequencies in *Txnrd2*^+/−^ L 2/3 PNs *in vitro*. (D) Comparison of mean dendrite and axon lengths in WT, *Txnrd2*^+/−^ and *LgDel* L 2/3 PNs *in vitro* (*n*=55 WT, 51 *Txnrd2*^+/−^, 36 *LgDel* L 2/3 PNs *in vitro*; one-way ANOVA, Holm−Šídák). (E) Basal respiration, spare capacity and ATP production differ in *Txnrd2*^+/−^ vs WT littermate control cultures (Seahorse; *n*=26 WT, 19 *Txnrd2*^+/−^ cultures/six litters; one-way ANOVA, Holm−Šídák). (F) Few significant differences, including DEGs, in *Txnrd2*^+/−^ vs WT L 2/3 PN transcriptomes (top; *n*=15 fetuses/five litters *Txnrd2*^+/−^; 15 fetuses/six litters WT). The two DEGs do not overlap *LgDel* vs WT. In addition, *Txnrd2* is diminished in *Txnrd2*^+/−^ L 2/3 PNs *in vitro*, validated by qPCR (bottom).

Acutely diminished *in vitro* or *in vivo* expression of the 22q11-deleted gene *Txnrd2* that encodes a rate-limiting enzyme for mitochondrial ROS clearance (Prasad et al., 2014; Soerensen et al., 2008) limits L 2/3 PNs dendrite and axon growth similar to that of the broader 22q11 deletion (Fernandez et al., 2019). To determine whether constitutive heterozygous *Txnrd2* deletion accounts for limited L 2/3 PN *in vitro* dendrite and axon growth, we cultured L 2/3 PNs obtained from E16.5 *Txnrd2*^+/−^ fetuses (Fig. 2A). Dendrite lengths of *Txnrd2*^+/−^ L 2/3 PNs *in vitro* were bimodally distributed (Fig. 2B, top); however, there was a uniform dendrite length shift towards ∼100 μm (Fig. 2C, top), resulting in intermediate mean dendrite length between WT and *LgDel* – significantly different from WT, but not *LgDel* (Fig. 2D, top). *Txnrd2*^+/−^ L 2/3 PNs *in vitro* axon lengths approximate those in WT (Fig. 2B,C, bottom); however, the larger axons that define the ‘tail' of the WT distribution were not seen in the *Txnrd2*^+/−^ L2/3 PNs *in vitro*. Mean *Txnrd2*^+/−^ axon length is also reduced compared to that in WT, but is greater than that in *LgDel*. None of these differences, however, are statistically significant (Fig. 2D, bottom). Thus, unlike acutely diminished *Txnrd2* expression *in vitro* and *in vivo*, constitutive heterozygous *Txnrd2* deletion did not substantially change the bimodal distribution of L 2/3 PN *in vitro* sizes or significantly limit L 2/3 PN *in vitro* axon growth.

**Fig. 3.**
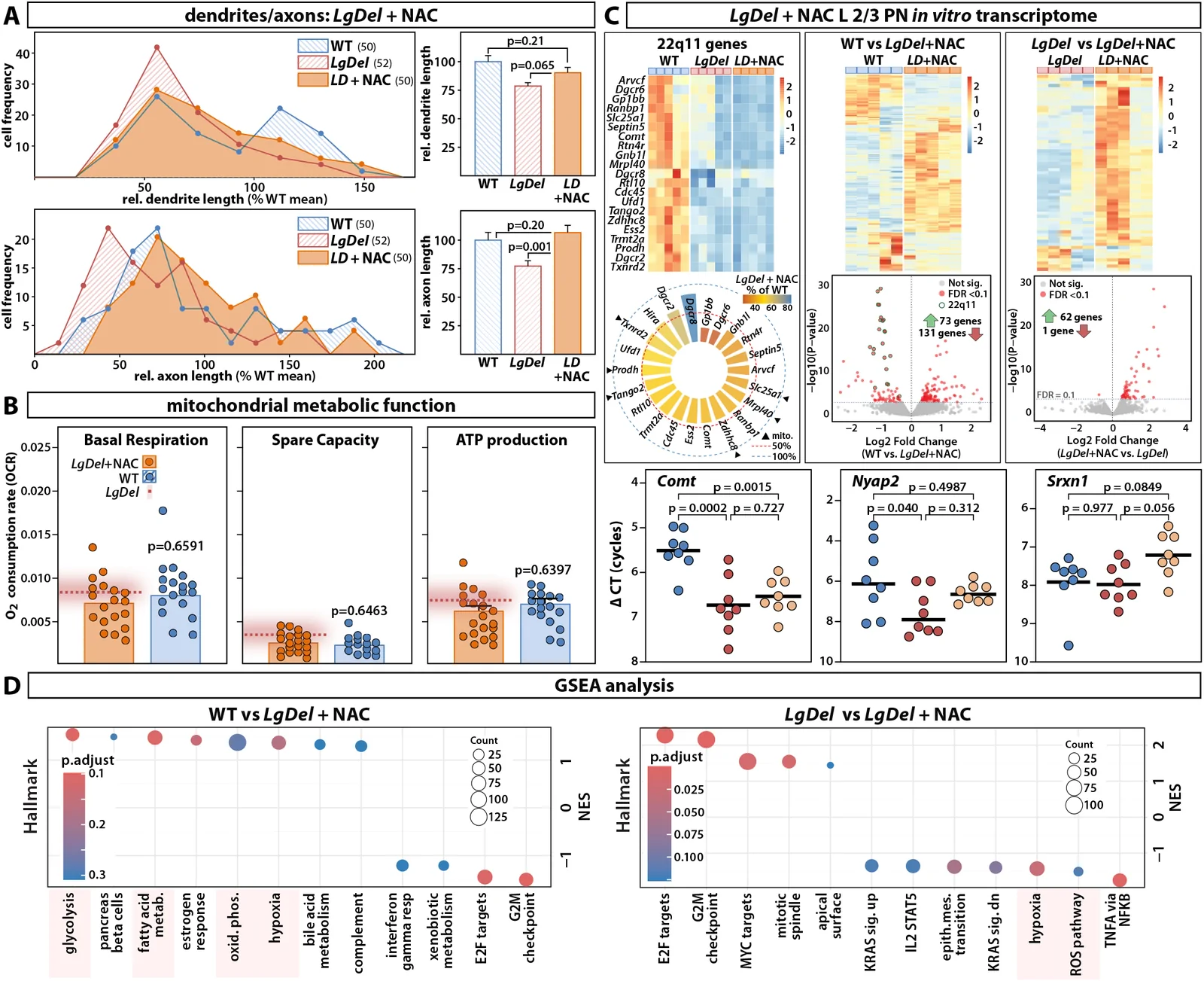
**Distinct cellular and transcriptional therapeutic responses to NAC increase dendrite and axon growth in L 2/3 PNs *in vitro*.** (A) NAC treatment yields a unimodal distribution of L 2/3 PNs *in vitro*; the mean increase in dendrite length does not return to WT bimodal distribution (top, left; data reanalyzed from Fernandez et al., 2019). *LgDel*+NAC L 2/3 PNs *in vitro* mean dendrite lengths are statistically indistinguishable from WT or *LgDel* (top, right). NAC restores the axon length distribution towards WT (bottom, left); mean length returns to one that is statistically indistinguishable from WT (bottom, right). (B) *LgDel*+NAC L 2/3 PN basal respiration, spare capacity and ATP production is statistically indistinguishable from WT littermate controls (*n*=17 cultures/four litters, one-way ANOVA, Holm−Šídák). (C) Transcriptome analysis of *LgDel*+NAC L 2/3 PNs *in vitro*. Twenty-one significantly expressed 22q11 genes (WT and *LgDel*) are diminished by 50% in *LgDel*+NAC L 2/3 PNs (top, left). The *LgDel*+NAC L 2/3 PN transcriptome differs from WT: there are 204 DEGs (top, middle) (*n*=15 fetuses/six litters WT; 15 fetuses/seven litters *LgDel*+NAC). Of 131 downregulated DEGs, 21 are 22q11 orthologues deleted in *LgDel*. When the *LgDel*+NAC L 2/3 PN *in vitro* transcriptome is compared to that of *LgDel*, none of the 64 DEGs overlap with DEGs from the WT vs *LgDel* comparison (*n*=15 fetuses/seven litters *LgDel*; 15 fetuses/seven litters *LgDel*+NAC). Validation of the antioxidant defense DEG *Srxn1* (bottom, left) by qPCR. Expression of *Nyap2*, a representative *LgDel* vs WT DEG that is significantly downregulated in *LgDel* L 2/3 PNs *in vitro* vs WT does not change significantly from WT levels in *LgDel*+NAC L 2/3 PNs *in vitro* (bottom, middle). (D) Hallmark GSEA of WT vs *LgDel*+NAC (top) and *LgDel* vs *LgDel*+NAC (bottom) transcriptomes. The WT vs *LgDel*+NAC comparison identifies multiple positively enriched gene sets associated with mitochondrial function (orange highlight; top). The *LgDel* vs *LgDel*+NAC comparison identifies negative enrichment for hypoxia and ROS gene sets (red shading), consistent with NAC antioxidant function.

Based upon apparent *Txnrd2* function in mitochondrial ROS clearance in L 2/3 PNs (Fernandez et al., 2019; Maynard et al., 2008), constitutive heterozygous *Txnrd2* deletion might alter L 2/3 PN *in vitro* mitochondrial metabolism as part of a response to altered L 2/3 PN *in vitro* growth. L2/3 PN *in vitro* O_2_ consumption diverges in *Txnrd2*^+/−^ vs WT littermate control L 2/3 PNs *in vitro* (Fig. 2E), unlike parallel measurements in *LgDel* and WT. Basal respiration, spare capacity and ATP production are lower in *Txnrd2*^+/−^ L 2/3 PNs *in vitro*. Differences for basal respiration and spare capacity approach statistical significance, and that for ATP production is marginally significant (*P*=0.0697, *P*=0.0639 and *P*=0.0549, respectively). Accordingly, mitochondrial function in the context of constitutive heterozygous *Txnrd2* deletion may diverge from WT and L 2/3 PNs *in vitro* with full deletion of 22q11 orthologues, including *Txnrd2*.

We next asked whether transcriptome changes similar to those in *LgDel* L 2/3 PNs *in vitro* accompany differences in *Txnrd2*^+/−^ L 2/3 PN *in vitro* growth and mitochondrial function. Aside from significantly diminished *Txnrd2* expression, the *Txnrd2*^+/−^ transcriptome does not change in parallel with that of *LgDel* L 2/3 PNs *in vitro* (Fig. 2F); instead, it is like WT. As in *LgDel*, *Txnrd2* expression, assessed by qPCR, declines by ∼50% in *Txnrd2*^+/−^ L 2/3 PNs *in vitro*. As expected, levels of other 22q11-deleted genes, including five additional mitochondrial genes, did not change (Fig. 2F, left). Finally, only two transcripts met statistical DEG criteria compared to WT: transcription of *GM8515*, which encodes a predicted protein of unknown function and is expressed at low levels in *Txnrd2*^+/−^ and WT L 2/3 PNs, is apparently diminished and transcription of *Saa2*, a serum amyloid protein expressed at variable levels, is apparently enhanced (Fig. 2F, right). Neither is differentially expressed in *LgDel* L2/3 PNs *in vitro*. Thus, constitutive heterozygous *Txnrd2* deletion does not substantially alter the transcriptional state of L 2/3 PNs *in vitro*.

Finally, to assess transcriptional changes independent of DEGs, we compared full *Txnrd2*^+/−^ (21,774 transcripts) and WT L 2/3 PN *in vitro* transcriptomes by using GSEA. The hallmark comparison between WT and *Txnrd2*^+/−^ transcriptomes yielded no significantly enriched gene sets, while that between *LgDel* and *Txnrd2*^+/−^ yielded enrichment in *LgDel* parallel to *LgDel* vs WT (see Fig. 1E). Thus, constitutive heterozygous deletion and reduced expression of *Txnrd2* limits growth and modifies metabolic state of L 2/3 PNs *in vitro* without substantial transcriptional changes, unlike a broader 22q11 gene deletion.

### 22q11-deleted L 2/3 PN *in vitro* growth and transcriptional response to NAC treatment

The ROS scavenger NAC belongs to a small group of mitochondria-targeted compounds that modify oxidative stress-related changes in 22q11DS animal models, human 22q11 gene-deleted iPSCs and individuals with 22q11DS (Crockett et al., 2025; Li et al., 2019). In *LgDel* L 2/3 PNs *in vitro*, NAC diminishes mitochondrial and cytosolic ROS, restores axon/dendrite growth and branching; in *LgDel* L 2/3 PNs *in vivo*, it enhances dendrite growth, reduces cytological signs of oxidative stress, increases synapse frequency and restores typical synapse morphology (Fernandez et al., 2019). We re-analyzed previous neuron growth data from WT and *LgDel* L 2/3 PNs *in vitro* with and without NAC, and confirmed the bimodal distribution of dendrite and axon lengths for WT L 2/3 PN *in vitro* (see Fig. 1), and their unimodal distribution for *LgDel* L 2/3 PNs *in vitro* (Fig. 3A). NAC does not fully restore *LgDel* L 2/3 PN *in vitro* dendrite or axon lengths to their WT distribution. Instead, their dendrite lengths distribute monotonically across larger values (Fig. 3A, top), whereas axon lengths approximate those of WT (Fig. 3A, bottom). A modest but significant dendrite/axon size correlation, less robust than that of WT, returns for *LgDel*+NAC L 2/3 PNs *in vitro* (Fig. S1). Mitochondrial metabolic function is unchanged from WT or *LgDel* L 2/3 PNs (Fig. 3B). This suggests that NAC affects a therapeutic adjustment that returns 22q11-deleted L 2/3 PNs *in vitro* to an enhanced – but not entirely typical – growth state.

NAC-dependent restoration of *LgDel* L 2/3 PN *in vitro* growth may either return *LgDel* DEGs levels to those of WT or elicit a unique transcriptional response that is independent of 22q11 genes or their downstream targets. As expected, all 21 of the 22q11-deleted genes expressed in *LgDel* L 2/3 PNs *in vitro* remained significantly diminished (Fig. 3C, top left); however, significantly DEGs identified by comparing *LgDel* and WT were also unchanged. The transcriptional state of *LgDel* L 2/3 PNs+NAC resembles neither WT nor *LgDel* (Fig. 3C). Of genes differentially expressed between *LgDel* vs *LgDel*+NAC L 2/3 PNs *in vitro*, 63 do not include any of the 29 non-22q11-deleted DEGs that distinguish *LgDel* L 2/3 PNs *in vitro* from WT (Fig. 3C, right). Instead, NAC-dependent DEGs include two upregulated transcripts: *Srxn1* and *Osgin1* associated with Nrf2-regulated antioxidant defense (Fig. 3C, right) (Brennan et al., 2017; Zhou et al., 2015). Thus, a distinct transcriptional response, rather than restoration to WT gene expression levels that had been altered by 22q11 gene deletion, accompanied enhanced dendrite and axon growth in 22q11 gene-deleted L 2/3 PNs *in vitro* following treatment with the antioxidant NAC.

Hallmark GSEA of the broader *LgDel*+NAC L 2/3 PN *in vitro* (19,730 transcripts) transcriptome vs *LgDel* or WT reinforces the impression that NAC elicits a novel transcriptional state to support metabolic homeostasis and mitochondrial function in 22q11-deleted L 2/3 PNs *in vitro* (Fig. 3D, top). Positive enrichment of one gene set, i.e. oxidative phosphorylation, is similar to that in the WT vs *LgDel* comparison (see Fig. 1E), albeit with lower significance. The *LgDel* vs *LgDel*+NAC comparison identifies negative enrichment for hypoxia and ROS (Fig. 3D, bottom), consistent with NAC antioxidant function relieving transcriptional correlates of oxidative stress in *LgDel* L 2/3 PNs *in vitro*. In addition, positive enrichment of hallmark gene sets of ‘E2F targets’ and ‘G2M checkpoint genes’ (negatively enriched in *LgDel* vs WT, see Fig. 1E) was consistent with NAC reversal of cellular stress that restricts growth or biases cells toward apoptosis. Apparently, NAC supports ‘therapeutic’ L 2/3 PNs *in vitro* growth in a novel transcriptional state that modulates antioxidant defense and circumvents growth-limiting effects of 22q11 deletion-dependent cellular stress.

### L 2/3 PNs have 22q11 deletion-specific growth and transcriptional responses to NAC

**Fig. 4.**
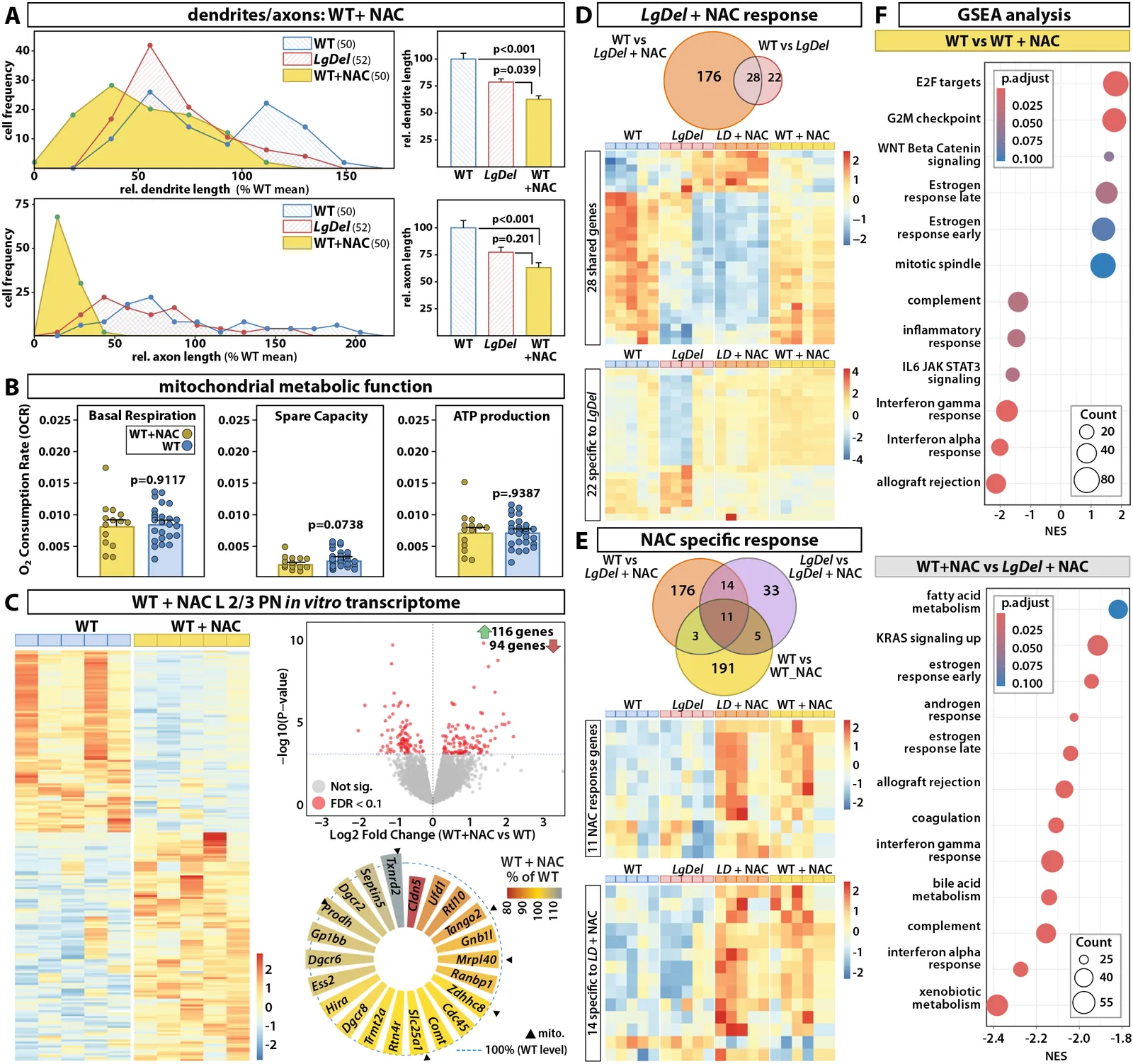
**NAC-regulated and 22q11-deletion-dependent NAC sensitive genes define a NAC-dependent therapeutic transcriptional response in 22q11-deleted L 2/3 PNs *in vitro*.** (A) Dendrite (top) and axon (bottom) frequency distributions in WT+NAC L 2/3 PNs *in vitro*. Growth is limited, and bimodal distribution (dendrites) or long ‘tails’ (axons) are no longer apparent (data re-analyzed from Fernandez et al., 2019). Mean WT+NAC dendrite and axon lengths are lower than WT and *LgDel* counterparts (right; *P*=0.0014 and 0.035; one-way ANVOA, Holm−Šídák). (B) WT+NAC L 2/3 PN *in vitro* metabolic function (O_2_ consumption) remains at levels comparable to WT and *LgDel* (see Fig. 1C). (C) Multiple DEGs in WT+NAC L 2/3 PNs *in vitro* vs WT counterparts (left) (*n*=15 fetuses/six litters WT; 15 fetuses/seven litter WT+NAC). Of 210 genes significantly differentially expressed in WT+NAC L 2/3 PNs *in vitro*, 116 were upregulated and 94 downregulated (top, right). 22q11-deleted genes are not among the DEGs (bottom, right). (D) 50 WT vs *LgDel* L 2/3 PN *in vitro* DEGs compared to 176 WT vs *LgDel*+NAC L 2/3 PN *in vitro* DEGs. Expression of 28 DEGs is sensitive to 22q11 deletion and NAC, while another 22 DEGs are regulated only by 22q11 deletion and insensitive to NAC. (E) Integration of WT vs *LgDel*+NAC, *LgDel* vs *LgDel*+NAC, and WT vs WT+ NAC L 2/3 PN *in vitro* transcriptomes (top) identifies 11 broadly NAC-responsive DEGs (shared across all comparisons) vs 14 DEGs that respond to NAC only in the context of 22q11 gene deletion. Heatmap comparisons confirm NAC-selective transcriptional changes broadly (11 DEGs, middle) or selectively for *LgDel*+NAC (14 DEGs, bottom). WT+NAC genes, expressed at similar levels to *LgDel*+NAC, are not significantly differentially expressed in the other comparisons. (F) Hallmark GSEA of WT vs WT+NAC (top) and WT+NAC vs *LgDel*+NAC transcriptomes.

The unique transcriptional response in NAC-treated 22q11-deleted *LgDel* L 2/3 PNs *in vitro* may encompass all or just a subset of genes that are also regulated by NAC in WT L 2/3 PNs *in vitro*. However, our previous observations indicate that NAC limits WT L 2/3 PN *in vitro* dendrite and axon growth (Fernandez et al., 2019). Indeed, the distribution of dendrite lengths in WT L 2/3 PNs *in vitro*+NAC resembled that in *LgDel* (Fig. 4A, top left) and mean dendrite length was moderately, but significantly, lower (Fig. 4A, top right). NAC substantially impacted WT axon growth. Axon lengths were distributed around a single mean that was less than that in the three other conditions (WT, *LgDel* and *LgDel*+NAC; Fig. 4A, bottom left). Nevertheless, NAC did not appear to alter mitochondrial metabolism in WT L 2/3 PNs; moreover, basal respiration, spare capacity or ATP production were statistically indistinguishable from those in WT (Fig. 4B). These results indicated that NAC limits WT L 2/3 PN *in vitro* growth and differentiation without disrupting these O_2_-consumption-based measures of mitochondrial function.

NAC may elicit a transcriptional response that inhibits dendrite and axon growth in WT neurons when ROS decline below typical levels (Mora-Zenil and Moran, 2024; Wilson et al., 2018) vs supporting *LgDel* L 2/3 PNs growth where NAC returns elevated ROS to WT levels. Thus, we compared the WT+NAC L 2/3 PNs *in vitro* transcriptome with their WT, *LgDel* and *LgDel*+NAC counterparts (Fig. 4C-F). Comparison of WT+NAC vs WT L 2/3 PNs *in vitro* (Fig. 4C, left) identified 210 DEGs of whom 116 were upregulated and 94 downregulated (Fig. 4C, top right). 22q11-deleted genes are not among these DEGs (Fig. 4C, bottom right). To determine whether the 50 DEGs that distinguish *LgDel* L2/3 PNs *in vitro* from WT were NAC-responsive or refractory, we compared WT L 2/3 PNs *in vitro* DEGs with *LgDel*+NAC or with *LgDel* alone (Fig. 4D, top). Shared differential expression of 28 transcripts common to WT vs *LgDel*+NAC and WT vs *LgDel* included 21 22q11-deleted genes and 22 *LgDel* vs WT DEGs (Fig. 4D, middle). These genes appear sensitive to the diminished 22q11 gene dosage but not NAC in *LgDel* L 2/3 PNs *in vitro*.

We next compared DEGs from WT vs *LgDel*+NAC, *LgDel* vs *LgDel*+NAC, and WT vs WT+ NAC to distinguish generally NAC-responsive genes from those that respond to NAC only in the context of 22q11 deletion. Eleven DEGs are shared across all three NAC-related pairwise analyses and, thus, are generally NAC responsive (Fig. 4E, top). These include (i) the antioxidant defense genes *Srxn1* and *Osgin1*; (ii) *Grm2*, encoding a glutamate receptor associated with neuronal activity and growth; (iii) *Wif1*, encoding a Wnt inhibitor associated with progenitor proliferation and axon growth, and (vi) *Rgs2*, encoding a protein associated with synaptic signaling and plasticity (Brennan et al., 2017; Heo et al., 2006; Hunter et al., 2004; Ingi et al., 1998; Ma et al., 2023; Soriano et al., 2008). These NAC-responsive DEGs were significantly upregulated in WT and *LgDel* NAC-treated L 2/3 PNs *in vitro*, while no significant changes were observed in either genotype without NAC (Fig. 4E, middle). In addition, 14 genes are responsive to NAC only in *LgDel* (Fig. 4E, bottom). These include *Nap1l5* and *Hdac9*, genes encoding proteins involved in nucleosome assembly and chromatin remodeling, and with essential roles in dendrite growth and synaptic maturation (Attia et al., 2013; Sugo et al., 2010), as well as *Adcyap1*, a gene that encodes adenylate cyclase activating polypeptide 1 and can promote neurite outgrowth and survival (Baskozos et al., 2020). Thus, when DEG sets are integrated a distinct set of therapeutic response genes emerges – some of which are generally NAC-responsive and others that respond to NAC only in the context of 22q11 deletion – to support dendrite and axon growth in 22q11-deleted L 2/3 PNs.

GSEA analyses of WT+NAC L 2/3 PNs *in vitro* (20309 transcripts) vs WT or *LgDel*+NAC L 2/3 PNs *in vitro* transcriptomes reinforce the impression that NAC treatment establishes a distinct transcriptional state to support WT L 2/3 PNs *in vitro* survival in the context of pathologically diminished ROS levels. Enriched gene sets include those of ‘E2F targets’, ‘G2/M checkpoint’ and ‘mitotic spindle’, all of which are associated with cell survival and growth (Fig. 4F, top). Unlike WT vs *LgDel* or *LgDel*+NAC (see Figs 1, 3); there is no significant enrichment of oxidative metabolism, stress or mitochondrial sets in WT vs WT+NAC. There is, however, negative enrichment of gene sets associated with inflammation (Fig. 4F, bottom), consistent with the anti-inflammatory properties of NAC (Askari et al., 2020; Santus et al., 2024). These data suggest that *LgDel* L 2/3 PNs *in vitro* have a unique transcriptional state that accommodates NAC-mediated ROS reduction and increased growth.

### Cellular and transcriptional states of 22q11-deleted L 2/3 PNs *in vivo*

**Fig. 5.**
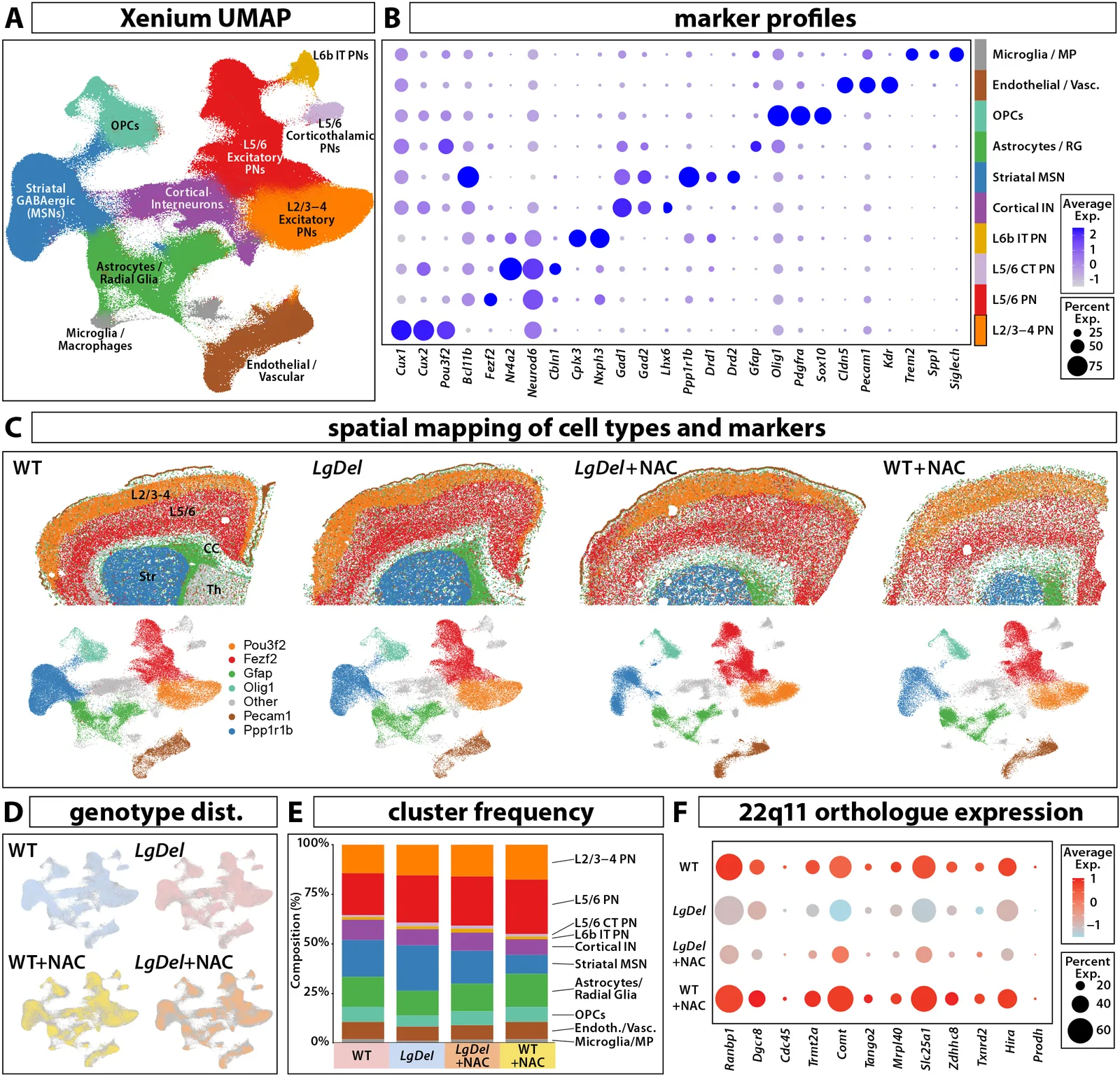
**Multiplex quantitative *in situ* spatial transcriptomic comparison of WT, *LgDel*, *LgDel***+**NAC and WT**+**NAC anterior forebrain, including L 2/3 PNs and their neighbors.** (A) Uniform manifold approximation and projection (UMAP) of forebrain cell types obtained by using principal component analysis (PCA) of cell type marker genes in the 347-probe panel identified ten distinct clusters that reflect identities of known forebrain cell types, including L 2/3 PNs and closely related L 4 counterparts, as well as two transcriptionally/connectionally distinct L 5/6 PN subclasses. (B) Distribution and expression magnitude of key marker genes across cell types derived from unbiased PCA analysis. (C) Cell types mapped onto representative WT (*n*=5 P6 mice, 1 section/mouse), *LgDel* (*n*=5 mice, 1 section/mouse), *LgDel*+NAC (*n*=5 mice, one section/mouse), and WT+NAC (*n*=3 mice, 1 section/mouse) sections (top row). PCA cell type localization is in register with known locations for each type. UMAPS for each genotype/treatment confirm that no cell classes are lost or gained across the four conditions (lower row). (D) Distributions of all cell clusters in each genotype/treatment condition (colors) correspond to the distribution of all cells analyzed together (gray). (E) Cell frequency/cluster is statistically indistinguishable across the four genotypes/conditions. Proportions of L 2/3-4 vs L 5/6 cells in *LgDel* and *LgDel*+NAC samples decline modestly, consistent with diminished L 2/3 PN frequency due to 22q11 deletion (Meechan et al., 2015b; Meechan et al., 2009). (F) Expression levels of 12 22q11-deleted genes in the 347-probe panel across each of four genotypes/conditions are reduced uniformly by ∼50% in *LgDel* and *LgDel*+NAC.

NAC reverses L 2/3 PN cellular pathology *in vivo* parallel to its *in vitro* activity (Fernandez et al., 2019). Thus, 22q11-deleted L 2/3 PNs *in vivo* may acquire a NAC-dependent transcriptional state that supports growth and differentiation parallel to L 2/3 PNs *in vitro*. To assess L 2/3 PN transcriptional responses that underlie the therapeutic effects of NAC *in vivo*, we first established that spatial transcriptomic RNA quantification *in situ* (Xenium; 10x Genomics Inc., Pleasanton, CA, USA) securely identifies L 2/3 PNs and their neighbors, thus, ensuring that transcriptional states can be assessed in intact cortices of early post-natal WT, *LgDel*, *LgDel*+NAC and WT+NAC L 2/3 mice. This approach, although limited by transcript numbers (347), nevertheless allowed reliable identification of all major forebrain cell classes (Fig. 5A; *n*=5 WT, *n*=5 *LgDel*, *n*=5 *LgDel*+NAC, *n*=3 WT+NAC mice at postnatal day 6 (P6); with one section per mouse analyzed). The relationship between expression levels of key markers, cell type(s) and neuroanatomical locations is robust across forebrain regions and cell classes, including upper (L 2/3-4) vs lower layer (L 5/6) cortical PNs (Fig. 5B,C). All cell types identified in the pooled uniform manifold approximation and projection (UMAP) (see Fig. 5A) are represented in each genotype/NAC treatment (Fig. 5C, bottom). No major cell class was lost or gained for any condition (Fig. 5D) and proportions are equivalent in each (Fig. 5E). Finally, a subset of 22q11-deleted genes added to the probe panel appears equally diminished across cell types in *LgDel* or *LgDel*+NAC vs WT or WT+NAC (Fig. 5F). Thus, multiplex hybridization/spatial transcriptomic analysis reliably identified cortical neuron classes for analysis of L 2/3 PN transcriptional changes in response to 22q11 deletion as well as NAC therapy *in vivo*.

**Fig. 6.**
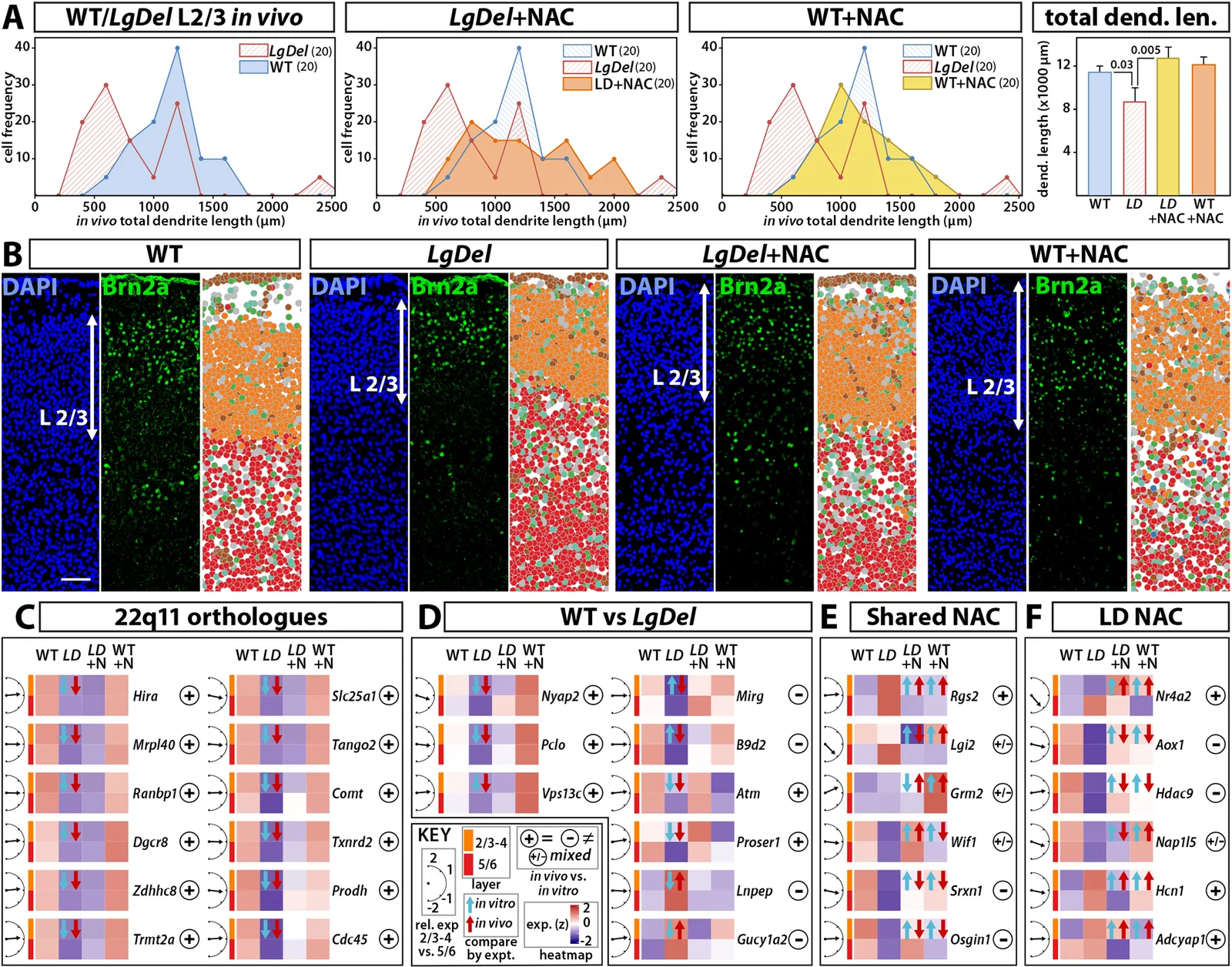
**L 2/3 PN *in vivo* transcriptional responses to 22q11 gene deletion and NAC treatment overlap with those of L 2/3 PNs *in vitro*.** (A) Distribution of total dendrite length/neuron for WT, *LgDel*, *LgDel*+NAC and WT+NAC L 2/3 PNs labeled *in vivo* by sparse Cux2^CreERT^ recombination (data re-analyzed from Fernandez et al., 2019). NAC restores *LgDel* mean dendrite length to WT levels and does not disrupt WT L 2/3 PN dendrite growth *in vivo* (far right). (B) Post hybridization immunohistochemical labeling for DAPI and Brn2a of WT, *LgDel*, *LgDel*+NAC and WT+NAC on the same Xenium sections (left and middle) used for spatial transcriptomic analysis (right) demonstrating integrity of cortical lamination and preservation of selective L 2/3 PN Brn2a expression, an established marker for L 2/3 PNs subset, and apparent diminished thickness and frequency of L 2/3 PNs in *LgDel* and *LgDel* + NAC sections. Scale bar: 50 µm. (C-F) Uniform diminished expression of 12 22q11-deleted genes in L 2/3 as well as L 5/6 PNs *in vivo*. Inset: a key for panels C through F. (C) The subset of DEGs that are heterozygously deleted in LgDel mice. (D) A subset of DEGs from the WT vs *LgDel* L 2/3 PN *in vitro* comparison are also expressed in L 2/3 PNs *in vivo*. (E) A subset of generally NAC-regulated DEGs identified in L 2/3 PNs *in vitro* (i.e. in *LgDel* + NAC and WT + NAC L 2/3 PNs *in vitro*) are expressed *in vivo*; however, expression changes in L 2/3 PNs *in vivo* do not fully accord with those *in vitro*. (F) A subset of DEGs identified as NAC-responsive only in the context of 22q11 deletion. Expression of one gene, Nr4a2 is substantially enhanced in L 5/6 vs L 2/3 PNs *in vivo*. L 2/3 PN expression changes *in vivo* for these genes accord generally with those *in vitro*.

To determine if *LgDel* L 2/3 PN dendrite growth *in vivo* is limited as *in vitro*, we compared total dendrite length distributions for WT and *LgDel* L 2/3 PNs *in vivo* based upon previously generated data (Fernandez et al., 2019). In contrast to those of L 2/3 PNs *in vitro*, WT L 2/3 PN *in vivo* dendrite lengths were distributed around a single mean while *LgDel* counterparts are distributed bimodally, i.e. a less frequent subset approximated the WT mean and a more frequent subset fell around a mean 50% of WT (Fig. 6A, left). NAC restores *LgDel* L 2/3 PN dendrite growth *in vivo* to a unimodal distribution (Fig. 6A, middle), and mean length was statistically indistinguishable from WT (Fig. 6A, furthest right). Unlike growth-limiting effects on WT L 2/3 PNs *in vitro*, NAC has no apparent influence on WT dendrite lengths *in vivo*. Thus, *in vivo* and *in vitro*, diminished 22q11 gene expression changed L 2/3 PN dendrite size distributions – albeit in distinct ways – and NAC enhanced growth without returning 22q11-deleted L 2/3 PN dendrites to a distribution similar to that for WT.

To assure that our transcriptome analysis identified L 2/3 PNs *in vivo*, we confirmed identity and location of a molecularly distinct subset by post-hybridization immunostaining (Fig. 6B). We observed an apparent decrease in *LgDel* L 2/3 thickness and L 2/3 PN frequency (Fig. 6B, center left), consistent with previous observations (Meechan et al., 2009, 2012; Rukh et al., 2026 preprint). Consistent with our *in vitro* data, NAC does not substantially modify L 2/3 or L 5/6 PN expression of 12 22q11-deleted genes in the Xenium probe set (Fig. 6B). In addition, the magnitude, as well as increased vs decreased gene expression, in L 2/3 PN *in vivo* vs *in vitro* is discordant for nine *LgDel* vs WT L 2/3 PN *in vitro* DEGs (Fig. 6C). In parallel, in L2/3 PNs *in vivo*, NAC generally modulated L 2/3 PN *in vitro* NAC-responsive (Fig. 6D) and *LgDel*-specific NAC-responsive genes (Fig. 6D,E); however, the magnitude and direction of *in vitro* vs *in vivo* expression changes diverged. Apparently, 22q11 and DEG expression that characterizes L 2/3 PNs *in vitro* (Fig. 3) is preserved *in vivo*; however, quantitative details of expression and response to NAC differ (Fig. 6C-F).

### A distinct mitochondria/metabolic transcriptional state in 22q11-deleted L 2/3 PNs *in vivo*

**Fig. 7.**
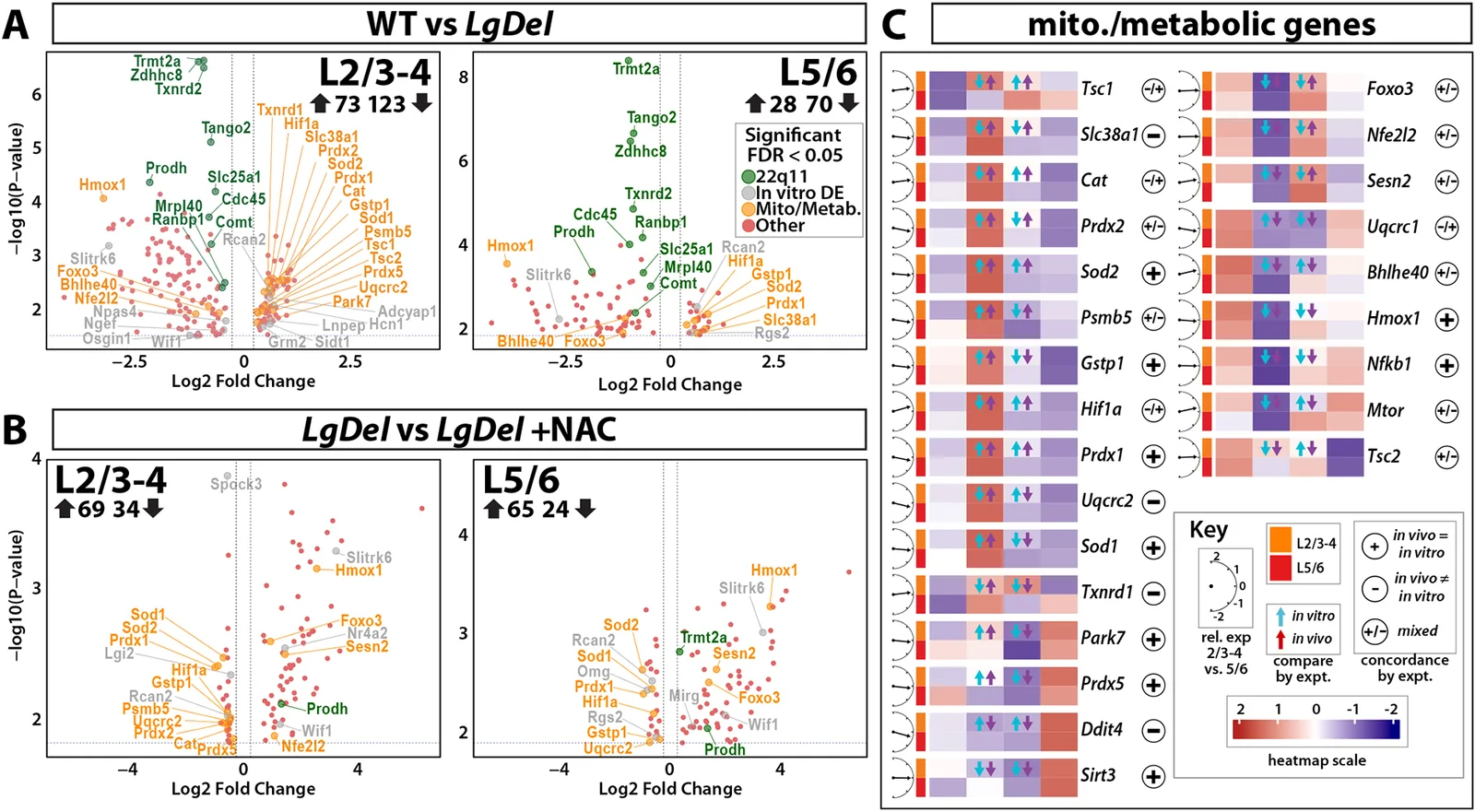
**22q11-deleted L 2/3 PNs *in vivo* have a distinctly different mitochondria/metabolic transcriptional state compared with their L 2/3 PN *in vitro* counterparts or L 5/6 PN *in vivo* neighbors.** (A) Volcano plots of DEG transcripts based upon pseudo-bulk seq for individual L 2/3 (left) vs L 5/6 PNs *in vivo* (right) from spatial transcriptomic data. Each plot shows down- and upregulated DEGs (left and right, respectively) of four key categories, i.e. 22q11-deleted genes (green dots), L 2/3 PN *in vitro* DEGs (gray dots), mitochondrial/metabolic regulatory/antioxidant defense/mitochondrial biogenesis and maintenance genes (orange dots), and additional genes in the probe panel (pink dots). (B) Volcano plots of significant DEGs in a pseudo-bulk seq comparison of *LgDel* vs *LgDel*+NAC L 2/3 (left) vs L 5/6 PNs (right) *in vivo*. The four categories of DEGs are as denoted in A. (C) Comparison of L 2/3 PN *in vivo* vs *in vitro* as well as L 5/6 PNs *in vivo* expression levels of the full set of 25 mitochondrial and metabolic regulatory genes chosen to assess oxidative stress, antioxidant defense, and their modulation by NAC. Relative expression levels in L 2/3 vs 5/6 PNs *in vivo*, as well as concordance or divergence of expression for each gene is indicated as in Fig. 6.

Our *in vitro* vs *in vivo* comparison, as well as previous observations (Fernandez et al., 2019), suggested that transcriptional changes in 22q11-deleted L 2/3 PNs are associated with mitochondrial and metabolic function. To further evaluate this possibility, we added Xenium probes for 25 regulators of mitochondrial integrity, metabolic homeostasis, and antioxidant defense to the standard probe panel for cell type identification. To assess potential differential regulation of these genes, we performed pseudo-bulk analysis in WT vs *LgDel* as well as *LgDel* vs *LgDel*+NAC based on L2/3-4 vs 5/6 PN cell classes identified by principal component analysis (PCA; see Figs 5, 6) to identify DEGs (FDR<0.05; Fig. 7A,B). There were more L 2/3-4 PN (73 upregulated, 123 downregulated) than L 5/6 PN (28 upregulated, 70 downregulated) DEGs in the WT vs *LgDel* comparison. More mitochondrial, metabolic regulatory and antioxidant defense genes were significantly differentially expressed in *LgDel* L 2/3-4 (19/25) (Fig. 7A, left) vs 5/6 PNs *in vivo* in the *LgDel* cortex (9/25) (Fig. 7A, right), and 15/19 (*LgDel*) were upregulated. A similar set of DEGs emerged for *LgDel* vs *LgDel*+NAC L 2/3 vs 5/6 PNs, respectively, i.e. 14 out of 25 mitochondrial, metabolic and antioxidant defense genes were significantly differentially expressed in *LgDel* L 2/3 PNs *in vivo* in response to NAC (Fig. 7B, left), and 9/25 were differentially expressed in L 5/6 PNs (Fig. 7B, right). Finally, variation in mitochondrial, metabolic and antioxidant defense gene expression – none of which are DEGs *in vitro* – is seen in L 2/3 PNs *in vitro*; however, direction and magnitude diverge (Fig. 7C). Apparently, NAC selectively reverses changes in L 2/3 PN mitochondrial metabolic regulatory and antioxidant defense gene expression *in vivo* without altering expression levels of 22q11-deleted or downstream DEGs. However, *in vivo* responses differed quantitatively from those of L 2/3 PNs *in vitro*.

## DISCUSSION

NAC antioxidant therapy reverses oxidative stress-associated developmental pathology due to diminished 22q11 gene dosage in a specific class of pyramidal neurons in the mammalian cerebral cortex neuron, i.e. cortical L 2/3 PNs, by circumventing deletion-associated changes in dendrite and axon growth as well as 22q11 deletion-dependent transcriptional changes *in vitro* or *in vivo*. NAC engages novel antioxidant defense mechanisms that support divergent, but beneficial, dendrite, axon and synapse growth as well as differentiation via 22q11 gene dosage-independent pathways. General features of this response are recognized in cultured primary 22q11-deleted L 2/3 PNs; however, *in vitro* vs *in vivo* changes diverge substantially. Thus, therapeutic mechanisms assessed *in vitro* – even in primary cultured neurons that retain several aspects of *in vivo* identity – likely require *in vivo* validation to define molecular pathways underlying therapeutic responses.

The loss of bimodal dendrite and axon sizes in 22q11-deleted L 2/3 PNs *in vitro*; inversion of this relationship *in vivo*, and failure of NAC to restore WT distributions *in vitro* or *in vivo* raises three questions. (1) Does diminished 22q11 gene dosage foreclose ‘maximal’ L 2/3 PN growth? (2) Does this reflect oxidative stress limited to differentiating L 2/3 PNs? (3) Could loss of maximal growth reflect altered L 2/3 PN genesis (Meechan et al., 2009, 2015b; Rukh et al., 2026 preprint)? If oxidative stress disrupts 22q11-deleted L 2/3 PN progenitor proliferation (Campbell et al., 2023; Paranjape et al., 2023; Purcell et al., 2023), diminishing ROS and enhancing antioxidant defense might stabilize genesis of L 2/3 PN neuroblasts capable of maximal growth. Alternatively, if such neuroblasts are lost due to divergent neurogenesis independent of oxidative stress, antioxidant therapy via NAC or other antioxidants, would be unlikely to restore WT L 2/3 PN dendrite or axon growth. These issues remain to be resolved for NAC-mediated therapeutic effects in developing L 2/3 PNs, and it is not yet clear whether NAC or other ROS scavengers can restore, maintain or enhance connections and function of mature 22q11-deleted L 2/3 PNs.

By analyzing differentiating L 2/3 PNs *in vitro* and *in vivo*, we have shown previously that acutely diminished *Txnrd2* expression *in vitro* or conditional heterozygous deletion of *Txnrd2 in vivo* recapitulates key aspects of L 2/3 PN pathology due to broader 22q11 deletion (Fernandez et al., 2019). In contrast, constitutive heterozygous *Txnrd2* deletion in developing L 2/3 PNs *in vitro* does not yield cellular, metabolic or transcriptional changes, making it unlikely that *Txnrd2* acts as a ‘contiguous gene’ to drive L 2/3 PN growth phenotypes associated with broader 22q11 deletion. The WT bimodal distribution of dendrite and axon arbor sizes remains despite diminished growth of *Txnrd2*^+/−^ L 2/3 PNs. Moreover, unlike broader 22q11 deletion, heterozygous mutation of *Txnrd2* alters mitochondrial metabolic activity assessed by O_2_ consumption. The extent to which antioxidants reverse possible metabolic pathology associated with constitutive heterozygous *Txnd2* mutation and its relevance to elevated ROS in 22q11-deleted L 2/3 PNs remains to be determined.

**Fig. 8.**
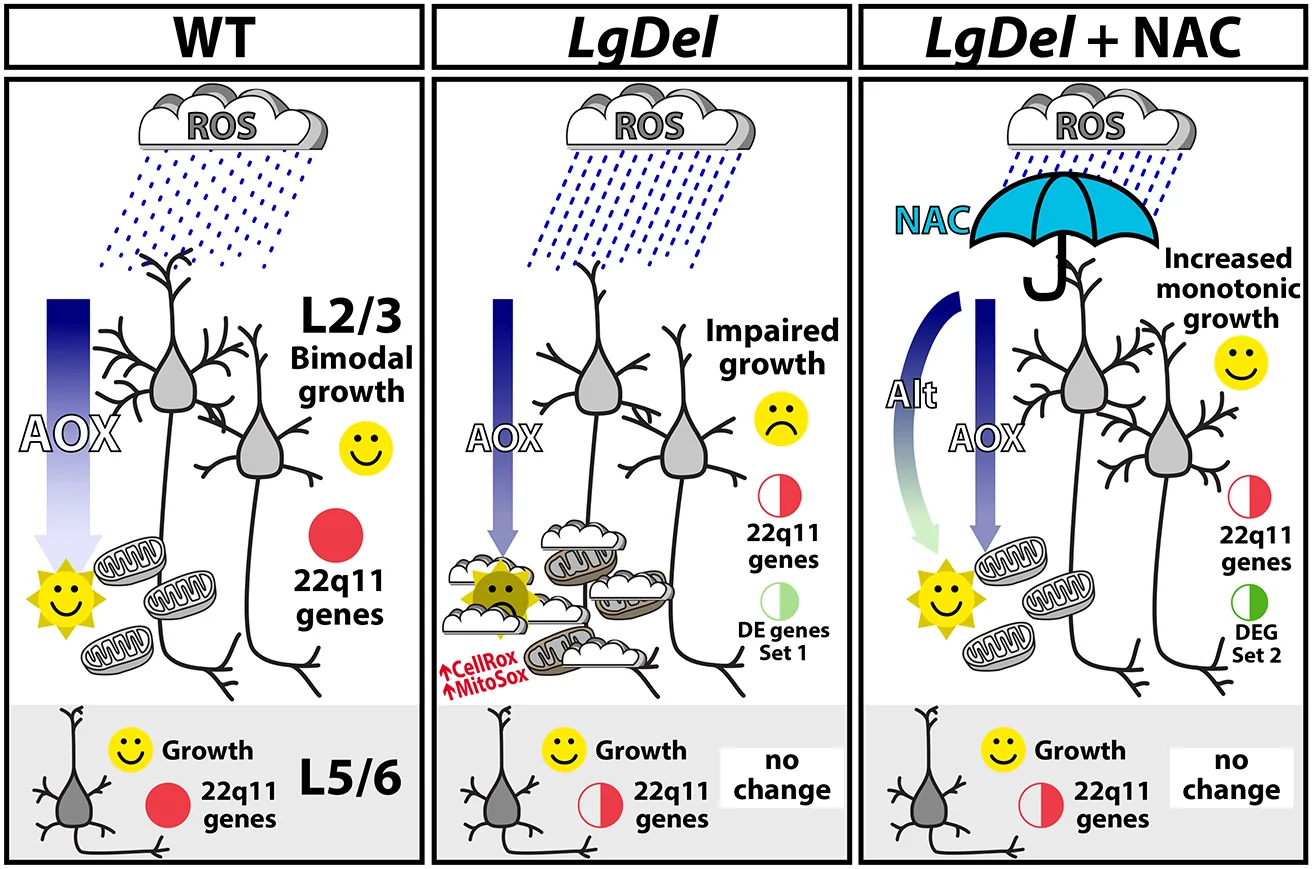
**NAC therapy selectively circumvents oxidative stress-mediated restricted growth in 22q11-deleted L 2/3 PNs by engaging distinct cellular and transcriptional responses.** Left panel: WT L 2/3 PNs (top) in the developing cortex vary in size and have unique metabolic/antioxidant defense transcriptional signatures that make them differentially sensitive to oxidative stress. L 5/6 PNs (bottom) do not share this metabolic/antioxidant defense signature. Middle panel: 22q11 deletion (LgDel) limits L 2/3 PN growth, especially of the largest WT L 2/3 PNs, and leads to mitochondrial changes due to increased ROS and resulting oxidative stress. These changes are not seen in L 5/6 PNs (bottom), despite diminished 22q11 gene expression in these neurons. Right panel: treatment with NAC, modifies diminished growth of L 2/3 PNs *in vitro* or *in vivo*, without returning them to the distribution of dendritic arbor sizes seen for WT. This therapeutically beneficial cellular response is limited to L 2/3 PNs *in vivo* and accompanied by a transcriptional response that restores neither 22q11 gene expression nor downstream genes that change their expression in response to diminished 22q11 gene dosage (red symbols). Instead, an alternative transcriptional response (green symbols) includes novel antioxidant defense genes that presumably support a divergent but salutary increase in L 2/3 PN growth and differentiation.

The ROS-scavenging activity of NAC does not reverse transcriptional consequences of 22q11 deletion in differentiating L 2/3 PNs *in vitro* or *in vivo*. Nevertheless, NAC – presumably by diminishing mitochondrial ROS and reducing oxidative stress in L 2/3 PNs (Fernandez et al., 2019) – elicits a novel transcriptional response that circumvents 22q11-deletion-dependent gene expression changes to support therapeutically beneficial but divergent L 2/3 PN growth and differentiation (Fig. 8). Thus, by identifying physiological states *in vitro* or *in vivo* – e.g. oxidative stress due to disrupted ROS clearance that underlies specific NDD developmental pathology, such as disrupted L 2/3 PN growth − may provide opportunities to exploit flexible cellular mechanisms and transcriptional networks as an alternative to direct functional restoration of mutant genes or restoration of their immediate downstream targets.

22q11-deleted L 2/3 PNs apparently occupy a unique oxidative environment during early post-natal association during cortico−cortical circuit development *in vivo*. These environmental and related NAC-mediated transcriptional changes are not shared by 22q11-deleted L 5/6 PNs *in vivo*. Our results suggest that laminar-selective oxidative stress in 22q11-deleted L 2/3 PNs and, perhaps, interneuron and glial neighbors (Lee et al., 2021; Shin et al., 2024; Steullet et al., 2017; Wang and Michaelis, 2010) drives association cortico−cortical circuit pathology, and its behavioral consequences in the *LgDel* 22q11DS mouse model. L 2/3-selective pathology may emerge only when bioenergetic demands to support L 2/3 PN growth and synaptogenesis increase during early post-natal life (Attwell and Laughlin, 2001; Chugani, 1998; Fernandez et al., 2019), or may have antecedents during L 2/3 PN genesis. frequency and identity of L 2/3 PNs are altered by divergent 22q11-deleted intermediate progenitors (Fenelon et al., 2013; Marinaro et al., 2017; Meechan et al., 2009; Paronett et al., 2015; Rukh et al., 2026 preprint). In contrast, L 5/6 PNs and their apical progenitors seem resilient, despite diminished expression of the same 22q11-deleted genes. It remains to be determined if L 2/3 vulnerability to metabolic stress is a pathogenic mechanism for multiple NDDs and, thus, amenable to mitochondria-targeted therapies (Frye et al., 2024; Khan et al., 2020; Pinto Payares et al., 2024; Rojas-Charry et al., 2021).

Our results using *in vitro* primary differentiating L 2/3 PNs and results of parallel studies that have used patient-derived or engineered iPSCs with 22q11 deletions (Liang and Zhang, 2013; Yoshihara et al., 2017) indicate that *in vitro* assays might require extensive adjustment to reliably identify 22q11DS pathogenic mechanisms and therapeutic targets. Variable results of several recent studies that have used engineered or patient-derived 22q11-deleted iPSC planar cultures, or cortical organoids amplified these challenges (Rao et al., 2025; Tegtmeyer et al., 2025). In iPSC planar cultures or organoids, as in primary murine cultures similar to those analyzed by us, some aspects of cellular and transcriptional identity are preserved; however, oxidative stress and other *in vitro* artifacts complicate alignment of *in vitro* and *in vivo* neuron classes (Bhaduri et al., 2020; Castiglione et al., 2025; Uzquiano et al., 2022). For 22q11DS, cortical interneurons or glial cells and systemic cardiovascular or immune changes (Cioffi et al., 2022; Crockett et al., 2021) might contribute to a uniquely aberrant *in vivo* L 2/3 metabolic environment with therapeutic targets not-yet identified *in vitro*. Understanding the balance of *in vitro* cell states and *in vivo* systemic influences will be key to optimize assays to investigate effective therapeutic agents in multiple polygenic NDDs (Eyring and Geschwind, 2021; LaMantia, 2024; Wang et al., 2022). Accordingly, *in vivo* validation of suggestive *in vitro* findings – especially in genomically and phenotypically accurate NDD animal models – is imperative to establish reliable mechanistic foundations for effective NDD therapies.

## MATERIALS AND METHODS

### Animals

All experimental procedures were approved by Virginia Tech Institutional Animal Care and Use Committee (#25-249). *LgDel* mice, carrying a hemizygous large deletion (LgDel) on chromosome 16 that spans the region between genes *Dgcr2* (bp: 17658219−17709592) and *Hira* (bp: 18695787−18789059), were maintained on a C57Bl/6N background. To minimize genetic drift, *LgDel* males were crossed with C57Bl/6N wild-type (WT) females (Charles River Laboratories). Timed matings were established by pairing *LgDel* males with WT females overnight; the morning of vaginal plug detection was designated as embryonic day (E)0.5. Pregnant dams were euthanized by rapid cervical dislocation at E16.5, and fetuses were collected in RNase-free HEPES buffer under sterile conditions. For postnatal experiments, natural deliveries were monitored daily, with the day of birth recorded as postnatal day 0 (P0); at P6 pups were collected for analysis. For genotyping, tail or limb samples were obtained from each embryo or pup and digested overnight at 60°C in SNET buffer (0.1 M Tris-HCl, 0.5 M NaCl, 0.1 M EDTA, 1% SDS, pH 8.0) supplemented with 0.5 mg/ml Proteinase K (Gold Biotechnology, St Louis, MO, USA). Genomic DNA was extracted using phenol/chloroform precipitation and used for PCR-based genotyping to identify WT and *LgDel* genotypes.

### Electroporation and primary neuronal culture

E16.5 embryos were collected from timed pregnant litters and dorsal telencephalic progenitors/early neuroblasts were transfected with pCI-GFP plasmid DNA (Addgene plasmid #87664) injected into the lateral ventricles (Fernandez et al., 2019; Hand et al., 2005). Following electroporation, cortices were dissected, dissociated and cultured for 5 days *in vitro* (DIV) as described (Ahlemeyer and Baumgart-Vogt, 2005; Fernandez et al., 2019). After 5 DIV, neurons exhibiting at least two distinct dendrites and a single axon measuring at least twice the length of the longest dendrite were selected for analysis. Neurons were reconstructed and traced (Imaris microscopy image analysis software, v10, Oxford Instruments, High Wycombe, UK) to quantify total dendritic and axonal length as well as branch numbers and branching order.

### NAC treatment *in vitro* and *in vivo*

N-acetylcysteine (NAC; supplied as PharmaNAC 900 mg tablets, BioAdvantex Pharma, Mississauga, ON, Canada) was administered at a final concentration of 1 mM during cell plating and replenished with each change of medium at 24 h (1 DIV) and 96 h (4 DIV). For *in vivo* experiments, NAC was delivered to pregnant dams by supplementing the drinking water with 900 mg/l NAC from gestational day 14 onwards. After birth, pups continued to receive NAC in their drinking water at the same concentration (900 mg/l), with fresh solutions prepared three times per week until tissue collection at P6 (Cabungcal et al., 2013; das Neves Duarte et al., 2012; Fernandez et al., 2019).

### Seahorse metabolic analysis

Primary cortical neurons were dissociated from the brain of mice at E16.5 as described above and plated at ∼1.4×10^5^ cells per well onto 96-well culture plates (Seahorse XFe96 FluxPak #103792, Agilent Technologies, Santa Clara, CA, USA). Each plate comprised cultures from a single litter, and L 2/3 PNs from each fetus/litter were cultured in triplicates (three wells/fetus). Cultures were maintained with changes of standard medium before being switched to XF DMEM Seahorse medium (#103680, Agilent) for metabolic assays. After a two-step equilibration, oligomycin, FCCP and rotenone/antimycin A were loaded per manufacturer's guidelines (Agilent SeaHorse MitoStress Test Kit, #103015), and oxygen (O_2_) consumption rate (OCR) was recorded via Agilent WAVE software. Following each experiment, cells were trypsinized from their culture wells and counted by using a hemocytometer to normalize data across wells. Basal respiration, spare capacity and ATP-dependent respiration were calculated as the mean from values obtained from each of the three wells representing each fetus. *LgDel*, *LgDel*+NAC, WT+NAC and WT data were derived from one independent set of experiments in which some cultures from each fetus were treated with NAC and others were not. The *Txnrd2*^+/−^ and littermate WT control data were derived from one independent set of experiments. The *n* numbers of fetuses/litters are provided in the relevant figure legend for each data set. Mean values for basal respiration, spare capacity and ATP-dependent respiration between genotypes/treatment conditions were compared using one-way ANOVA with Holm−Šídák multiple comparison correction.

### RNA sequencing

RNA was extracted from DIV5 cultures of WT and *LgDel*, Txnrd2^+/−^, WT+NAC, *LgDel*+NAC groups. At least five biological replicates were sequenced for each genotype/condition, with each biological replicate representing pooled RNA from at least three (typically five) embryos from a least three different litters and distinct culture experiments. Isolated RNA with RNA integrity number (RIN) scores of 10 (Agilent Tapestation 4200, Virginia Tech Genomics Core, Blacksburg, VA, USA) were pooled for each replicate. Libraries were generated and sequenced on an Illumina HiSeq platform (Novaseq 6000, Genewiz, South Plainfield, NJ, USA), yielding a depth of ≥90 million paired-end 150-bp-long reads per sample. Read quality was assessed with FastQC (https://www.bioinformatics.babraham.ac.uk/projects/fastqc/), and trimming/filtering was performed with BBDuk (https://github.com/BioInfoTools/BBMap/blob/master/sh/bbduk.sh). Reads were aligned to the GRCm38/mm10 genome using (Spliced Transcripts Alignment to a Reference (STAR; https://github.com/alexdobin/STAR), achieving a minimum unique alignment rate of 85%. Transcript abundance was quantified as transcripts per million (TPM). Differential gene expression analysis was performed using edgeR in R (https://bioconductor.org/packages/release/bioc/html/edgeR.html). Low-count genes (<15 reads in ≥3 samples) were filtered out. Data were normalized, and a robust dispersion estimation was used. Differentially expressed genes (DEGs) were identified by using a likelihood ratio test [false discovery rate (FDR) cut-off of <0.1]. GSEA analysis was performed using the R package of the Fast Gene Set Enrichment Analysis (FGSEA) method (https://bioconductor.org/packages/release/bioc/html/fgsea.html; Korotkevich et al., 2021). Gene ontology enrichment for DEGs was performed in Metascape (https://metascape.org/gp/index.html#/main/step1) using default settings (Zhou et al., 2019).

### Histological preparation

P6 pups were anesthetized by urethane injection, then transcardially perfused and post-fixed overnight in 4% paraformaldehyde in PBS. Following cryoprotection (sequential overnight immersion in 10%, 20% and 30% sucrose solution in PBS at 4°C), brains were embedded in OCT (Optimal Cutting Temperature compound, Fisher Healthcare, Waltham, MA, USA) in a consistent orientation and stored at −80°C; 12-μm coronal cryostat sections were mounted on slides, dried at room temperature, and stored at −80°C.

### Real-time quantitative (q)PCR

cDNA synthesis reactions were conducted as previously described (Maynard et al., 2013) on RNA isolated from primary cultures. qPCR for multiple transcript-specific primers was performed on a CFX384 thermal cycler using EvaGreen (Bio-Rad).

### Xenium spatial transcriptomic analysis

Fixed frozen tissue sections of 12 μm thickness (prepared as above on Xenium slides) were processed according to the 10x standard procedure described by Janesick et al. (2023). Xenium spatial sequencing data for a customized 347-probe panel were analyzed using the Xenium platform (10x Genomics Inc., Pleasanton, CA, USA (https://www.10xgenomics.com/support/software/xenium-ranger/downloads), and subsequent processing was performed in R (v4.5; https://cran.r-project.org/) with the Seurat (v5.1.0; https://github.com/satijalab/seurat; Butler et al., 2018) and SeuratDisk packages (https://github.com/mojaveazure/seurat-disk). Raw outputs were imported via LoadXenium() and filtered for low quality cells based on number of transcripts (nCount) and number of expressed genes (nFeature). Expression counts were log-normalized using the NormalizeData() function; variable features were identified via FindVariableFeatures using the ‘vst' method and canonical cell type markers to annotate clusters (Zeisel et al., 2015). Dimensionality reduction was performed by principal component analysis (PCA) followed by uniform manifold approximation and projection (UMAP) for visualization. The dataset was clustered using the Louvain algorithm [FindNeighbors() and FindClusters()] at a resolution of 0.1, resulting in ten transcriptionally distinct clusters. Spatial visualization was generated using the SpatialDimPlot() and SpatialFeaturePlot() functions, overlaying cluster or gene expression information on the section image. Additional spatial analyses, including expression plots and marker gene identification, were performed using FindAllMarkers() with the Wilcoxon rank-sum test. Cell proportional analysis was performed using ANOVA with Dunnett’s post hoc test, comparing all conditions to WT controls. Across all conditions 347 genes were assessed for differential expression by using EdgeR in R (Robinson and Smyth, 2007). All analyses were performed on a high-performance workstation (Intel-Xeon Silver 4208 CPU, 16 threads, 256 GB RAM). Brain sections from Xenium experiments were stained and imaged as described in previous study (Rukh et al., 2026 preprint).

### Statistics

Comparisons of neuronal growth metrics and mitochondrial respiration parameters across genotypes and treatment conditions were performed by one-way ANOVA with Holm-Šidák correction for multiple comparisons. Pearson correlation was used to assess the relationship between dendrite and axon length within individual neurons. These analyses were performed in Prism (version 10, GraphPad Software, Boston, MA, USA); significance was set at α=0.05. Statistical approaches specific to RNA sequencing and spatial transcriptomic analyses use the approaches described in the relevant Materials and Methods subsections above.

### AI usage

ChatGPT was used for code refactoring; all AI generated code was proofed and tested for functional validity before use as an analytical tool.

## Supplementary Material

10.1242/dmm.052786_sup1 Supplementary information
