# Supplementary material for: An antioxidant therapy elicits distinct transcriptome responses in 22q11-deleted upper layer cortical projection neurons
Source: Dis Model Mech. 2026 Sep 9;19(9):dmm052786. doi: 10.1242/dmm.052786 (PMC13602371; doi:10.1242/dmm.052786)
Supplement: Supplementary file 1 [file dmm-19-052786-s1.pdf]

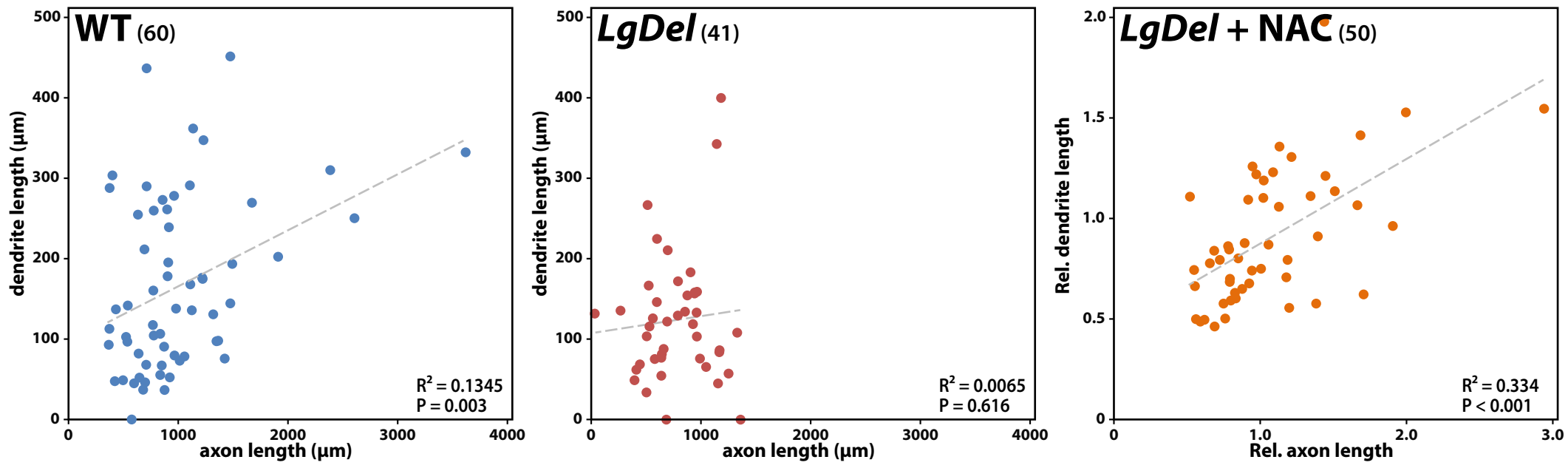

Fig. S1. Correlations between dendrite and axon sizes per cell for WT, LgDel, LgDel + NAC L 2/3 PNs *in vitro*. These correlograms are based upon data plotted for dendrite and axon sizes in main Figures 1 and 3.

Disease Models & Mechanisms • Supplementary information
